# A hybrid metaheuristic-deep learning technique for the pan-classification of cancer based on DNA methylation

**DOI:** 10.1186/s12859-022-04815-7

**Published:** 2022-07-11

**Authors:** Noureldin S. Eissa, Uswah Khairuddin, Rubiyah Yusof

**Affiliations:** 1 Department of Computer Engineering, Arab Academy for Science, Technology and Maritime Transport, Cairo, Egypt; 2 Centre for Artificial Intelligence and Robotics, Malaysia-Japan International Institute of Technology, Universiti Teknologi Malaysia, Kuala Lumpur, Malaysia; 3 Department of Mechanical Precision Engineering, Malaysia-Japan International Institute of Technology, Universiti Teknologi Malaysia, Kuala Lumpur, Malaysia

**Keywords:** Cancer, Deep learning, DNA methylation, Metaheuristic, TCGA

## Abstract

**Background:**

DNA Methylation is one of the most important epigenetic processes that are crucial to regulating the functioning of the human genome without altering the DNA sequence. DNA Methylation data for cancer patients are becoming more accessible than ever, which is attributed to newer DNA sequencing technologies, notably, the relatively low-cost DNA microarray technology by Illumina Infinium. This technology makes it possible to study DNA methylation at hundreds of thousands of different loci. Currently, most of the research found in the literature focuses on the discovery of DNA methylation markers for specific cancer types. A relatively small number of studies have attempted to find unified DNA methylation biomarkers that can diagnose different types of cancer (pan-cancer classification).

**Results:**

In this study, the aim is to conduct a pan-classification of cancer disease. We retrieved individual data for different types of cancer patients from The Cancer Genome Atlas (TCGA) portal. We selected data for many cancer types: Breast Cancer (BRCA), Ovary Cancer (OV), Stomach Cancer (STOMACH), Colon Cancer (COAD), Kidney Cancer (KIRC), Liver Cancer (LIHC), Lung Cancer (LUSC), Prostate Cancer (PRAD) and Thyroid cancer (THCA). The data was pre-processed and later used to build the required dataset. The system that we developed consists of two main stages. The purpose of the first stage is to perform feature selection and, therefore, decrease the dimensionality of the DNA methylation loci (features). This is accomplished using an unsupervised metaheuristic technique. As for the second stage, we used supervised machine learning and developed deep neural network (DNN) models to help classify the samples’ malignancy status and cancer type. Experimental results showed that compared to recently published methods, our proposed system achieved better classification results in terms of recall, and similar and higher results in terms of precision and accuracy. The proposed system also achieved an excellent receiver operating characteristic area under the curve (ROC AUC) values varying from 0.85 to 0.89.

**Conclusions:**

This research presented an effective new approach to classify different cancer types based on DNA methylation data retrieved from TCGA. The performance of the proposed system was compared to recently published works, using different performance metrics. It provided better results, confirming the effectiveness of the proposed method for classifying different cancer types based on DNA methylation data.

**Supplementary Information:**

The online version contains supplementary material available at 10.1186/s12859-022-04815-7.

## Background

Cancer is a growth of tissue that originates from an abnormal division of eukaryotic cells that eventually destroy normal surrounding tissues. It is soon expected to top the list of non-communicable diseases, and currently, one-sixth of all deaths worldwide are caused by cancer [1]. Therefore, it is very important to help find and develop techniques to detect and treat it. One of the major challenges in doing so is that cancer can mimic other diseases [2–4], which hinders a lot of effort trying to diagnose it using traditional methods that rely on differential diagnosis and involve many medical scans and tests that are often very expensive. Diverse factors like genetics, viruses, and environmental agents cause such malignancy; at the same time, newer ones are frequently being discovered.

DNA methylation is an epigenetic process that regulates gene expression without altering the DNA sequence. It is widely believed to be a key to better cancer diagnosis [5, 6]. It can be used to study different gene functions that are otherwise incomprehensible using traditional alterations in DNA sequences. DNA methylation is dynamically affected by various external factors such as environmental risks, and internal factors such as complex disease pathology [7]. In mammals, such as humans, DNA methylation works by transferring a methyl group onto the C5 position of the cytosine to form 5-methylcytosine, where the gene expression is regulated by using proteins involved in gene repression or by inhibiting the binding of transcription factor to the DNA [8].

New technologies that employ next-generation sequencing (NGS) and microarrays, such as those provided by Illumina Infinium [9, 10], can provide very high throughput with relatively low prices, especially since they can be reused many times on different patients. Enormous data availability helped advance cancer associated research because much valuable information could be extracted. Yet at the same time, it created a massive problem concerning the application of traditional data mining and analysis techniques [11]. The Human Genome Project indicates that approximately $30\times 10^6$ CpG dinucleotides can exist in methylated or unmethylated states. Therefore, the possible combinations of methylation arrangements are enormous. The work proposed in this research relies on big data techniques, specifically metaheuristic approaches [12–14] to decrease data dimensionality, followed by deep learning techniques to help classify the different types of cancers.

### Liteature review

In this regard, Akalin et al. [15] proposed MethylK, a software package written in R-language to analyse DNA methylation data using unsupervised learning methods to extract useful information. They demonstrated the software capabilities using breast cancer samples. While the software package is multi-threaded, it can only operate on a single machine, which negatively impacts its performance for massive datasets that involve pan classification of various cancer diseases.

Celli et al. [16] introduced BIGBIOCL, an algorithm that uses a supervised classification technique to extract relevant features from DNA methylation data. The algorithm was tested using data on three different types of cancers retrieved from the Cancer Genome Atlas (TCGA). The suggested technique used 70% of the data to build the classification model, while 30% was reserved for testing. The proposed model worked iteratively, where a set of new features (genes) is added with each iteration. However, since Fabrizio Cellia et al. did not intend to build the model to classify new data, it can be readjusted to build a classification model using 100% of the input to further validate the results. It should be noted that the authors’ primary target was to extract the relevant cancer features rather than classifying cancer types.

Recently, Zheng et al. [17] proposed a deep neural network (DNN) model that can work with DNA methylation data to predict cancer origins based on data retrieved from the cancer genome atlas (TCGA). Their method included a feature selection technique that can remove the noise in the data. The authors used a one-way analysis of variance (ANOVA) to filter the CpG methylation sites with very similar beta values (p >0.01) for different tissues, resulting in 18,976 CpG sites. Next, the authors applied Tukey’s honest significance difference test to remove the CpG locations with maximal mean beta values of less than 0.15. Ultimately, they were able to identify 10,360 CpG sites that acted as the input for the deep learning model. The multilayer perceptron (MLP) was used to construct the deep neural network. The results have shown excellent potential in diagnosing cancer of unknown primary origin and identifying circulating tumour cells. Their work helped prove that DNN models for DNA methylation have great potential in diagnosing cancer of unknown origins and detecting cancer cell types related to circulating tumour cells.

In 2021, Modhukur et al. [18] used different machine learning approaches to classify primary and metastasised cancers using DNA methylation samples retrieved from TCGA and other sources. They applied Support Vector Machines (SVM), Extreme Gradient Boosting, Naive Bayes (NB), and Random Forest (RF) approaches to classify the cancer types. They achieved the highest average accuracy of 99% using the RF method.

By inspecting the recent relevant research in the literature, it seems that the performance and scalability of the implemented methods are major challenges, especially since we are dealing with huge datasets. Moreover, challenges also arise because the DNA methylation mechanism and its relation to cancer are not currently well understood and are still being investigated [19]. Also, the literature focuses on classifying individual cancer types rather than pan classification. The work proposed in this research tackles these issues using a scalable solution that supports multi-threading and multiple host capabilities to implement feature extraction. Since the DNA methylation mechanism is not yet well understood; the proposed system first implements an unsupervised metaheuristic technique that performs feature selection, then it builds a supervised classification model based on a deep neural network (DNN) to classify cancer types based on the malignancy information found in the data.

## Methodology and tools

The proposed system encompasses two main stages. The first stage is based on an unsupervised metaheuristic technique that implements the feature selection and reduces dataset dimensionality. The second stage of the system is a deep learning pan classification model. This chapter presents the dataset and demonstrates the proposed system.

### Datasets

The proposed technique is tested using real-world datasets built using data collected from the cancer genome atlas (TCGA) project. The most critical measure that reflects the level of DNA methylation is known as the beta value. The DNA methylation file (for each sample) includes a column that contains the beta values and rows that reflect the various CpG locations. The CpG locations are the features that we need to reduce. The beta value for each CpG site is calculated as follows [20]

1 $$\begin{aligned} \beta _n = \frac{Max(M_n, 0)}{Max(M_n,0) + Max(U_n,0) + \alpha } \end{aligned}$$

where $M_n$ and $U_n$ are the methylated and unmethylated gene intensities, at location n. It is also worth noting that the beta value ranges from 0 to 1, where a value of 0, under perfect conditions, means that no methylated molecules were detected at these CpG sites, and a value of 1 indicates that all molecules were completely methylated. The $\alpha$ value is a constant offset added to the denominator to calibrate the beta value when both the unmethylated and the methylated values at a given location have very low intensities. Illumina recommends this value for their DNA methylation assays, and it has a default value of 100.

To test the proposed system, a basic pilot study that uses the three types of cancer diseases listed in Table 1 was conducted. These samples are synthesized using the Illumina Infinium 27k metyhlation platform. More information about the samples can be found in files [Breast.csv, Ovary.csv and Stomach.csv] in the additional files section.

**Table 1:** Cancer samples based on Illumina Infinium 27k metyhlation platform

Cancer name	TCGA project	Number of samples	Number of malignant samples	Cancer type
Breast	TCGA-BRCA	342	312	Breast invasive carcinoma
Ovary	TCGA-OV	613	567	Ovarian serous cystadenocarcinoma
Stomach	TCGA-STAD	73	47	Stomach adenocarcinoma

To further test the scalability and performance of the proposed method, another study that encompasses more cancer diseases and that uses samples profiled using the superior Illumina Infinium 450k metyhlation platform, was conducted. Table 2 lists the cancer diseases for the second study. Information about these samples can be found in files [Breast450.csv, Colon450.csv, Kidney450.csv, Liver450.csv, Lung450.csv, Prostate450.csv and Thyroid450.csv] in the additional files section.

**Table 2:** Cancer samples based on Illumina Infinium 450k metyhlation platform

Cancer name	TCGA project	Number of samples	Number of malignant samples	Cancer type
Breast	TCGA-BRCA	596	499	Breast invasive carcinoma
Colon	TCGA-COAD	344	307	Colon adenocarcinoma
Kidney	TCGA-KIRC	439	319	Kidney renal clear cell carcinoma
Liver	TCGA-LIHC	258	209	Liver hepatocellular carcinoma
Lung	TCGA-LUSC	291	249	Lung squamous cell carcinoma
Prostate	TCGA-PRAD	399	349	Prostate adenocarcinoma
Thyroid	TCGA-THCA	454	398	Thyroid carcinoma

The data was retrieved from the cancer genome atlas (TCGA) portal using custom-written software, since at the time of writing this manuscript, the portal did not allow batch download of samples. The retrieved data were first processed to handle some missing methylation values, since the microarray technology is always susceptible to some noises and the data almost always suffer from losses.

A linear regression method is considered reliable for this task and was used to interpolate the missing values and handle the situation [21]. DNA methylation samples with more than 10% and 20% of missing values for 27k profiles and 450k profiles, respectively, were rejected and removed from the training process.

The final dataset for each cancer type was built by transposing all the CpG locations for each sample into columns. By repeating the process for all the samples, we have a dataset that contains a massive matrix in which the rows correspond to different samples, and the columns represent the various CpG beta values.

### Unsupervised metaheuristic feature selection

The proposed metaheuristic technique uses evolutionary learning algorithms to perform feature selection, hence, reduce data dimensionality. In the field of machine learning, evolutionary learning algorithms [22, 23] are search-based techniques that can be utilised to solve optimisation problems. As previously highlighted, the CpG methylation process suffers from incomplete biological comprehension. Therefore, we think selecting metaheuristic techniques to approach this problem is more realistic because of its superior global search capabilities. The proposed metaheuristic system is based on genetic algorithms (GAs) [24], where two GAs work together to extract the features. The GAs are designed to work in a nested hierarchy, where the fitness function of the inner GA layer is passed to the outer layer.

One GA performs unsupervised clustering by taking advantage of the data’s mathematical similarities and elemental structure, while the other GA extracts features. The unsupervised clustering technique is only used to evaluate the selectivity of the features. This nested hierarchy sets the fitness of the clustering GA as a crucial feedback (multiplied by selected features) to the feature selection GA. The ultimate task for the clustering GA is not to explicitly achieve a clustering configuration; instead, it is to use an appropriate clustering separability measurement that reflects better feature selection.

At first, a random group of features are selected to cluster the data, and then the separability of the resulting clusters is evaluated using the proposed fitness function. Therefore, better separation of clusters signifies better feature selection and noise elimination. It is crucial to note that DNA methylation data are not only limited to cancer. So, the final data for this stage must only include DNA methylation data for patients with an established cancer history. It is to ensure that the unsupervised clustering technique would find similarities across the different samples. Chromosome size is based on the number of samples for the data clustering GA. In the beginning, the chromosome is divided into logical clusters with random sizes and count. Throughout the convergence process, the clusters start to form optimum partitions. The genes are integers representing each patient (sample) from the dataset. During the convergence process, the different CpG beta values for each patient are fetched from the cache and used for the calculations.

Two setups were initially considered for the GAs. The first setup uses the clustering GA as the outside layer. The chromosome is initialised with random clusters encompassing all the existing features in this scenario. For each of these chromosomes, a complete fork of feature selection GA (inner layer) will be executed, which will then modify the features of the clustered data. The second setup swaps the clustering and the feature selection GAs. The outer layer chromosomes will be initialised with random binary features in this scenario. The inner layer clustering GA will be executed to cluster the data based on these selected features. The fitness value is calculated based on cluster separability and is explained further on. Since the logical clusters’ configuration are set by each chromosome, the number of clusters does not need to be set a priori, as this is expected to reach a near optimum value with the convergence of the GA.

**Fig. 1:**
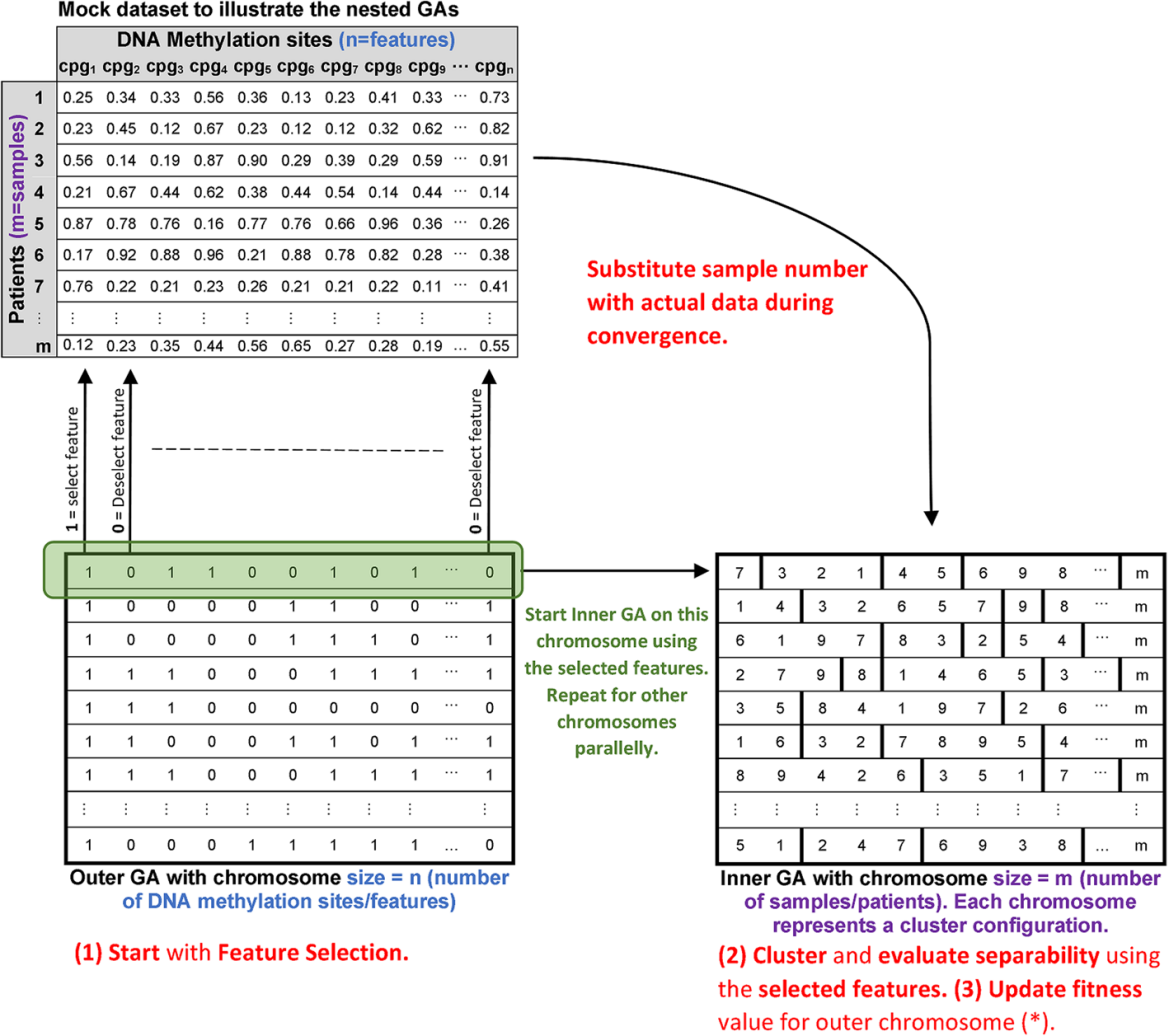
Illustration of the selected nested GAs architecture

The second setup, illustrated in Fig. 1, was selected since as evident from the literature, clustering the DNA methylation data without decreasing unnecessary features is computationally intensive. Also, data clustering without prior feature selection will produce inferior results because insignificant features are considered as noise during the clustering process [25, 26].

#### Outer layer genetic algorithm (OLGA)

The outer layer GA performs the feature selection based on the inner layer GA feedback. For the crossover, a standard single-point crossover operator was implemented. Selection is made based on a roulette-wheel operator. Since this is a simple binary-based GA, the crossover only swaps the genes on both ends of the selected cut-off point. The mutation operator was non-uniform, and its probability was calculated based on the best and the average fitness of the latest generation. The maximum probability for the mutation operation was set to 5%, and it worked by flipping the bits. The selected features were assigned a value of '1’ and passed to the clustering layer, while '0’ was assigned to unselected features.

Elitism was also used to copy the fittest individual to the new generation. As already explained, the fitness value of the outer layer GA was not independently calculated. Instead, it was retrieved from the inner GA layer after convergence. This is crucial to create a feedback mechanism between the separability of the clusters and the selectivity of features.

#### Inner layer genetic algorithm (ILGA)

The inner layer genetic algorithm is mainly used to evaluate the fitness of the selected features assigned from the outer GA layer. It is done by clustering the data and evaluating their separability, where better separability means a better selection of features and superior noise elimination.

**Fig. 2:**
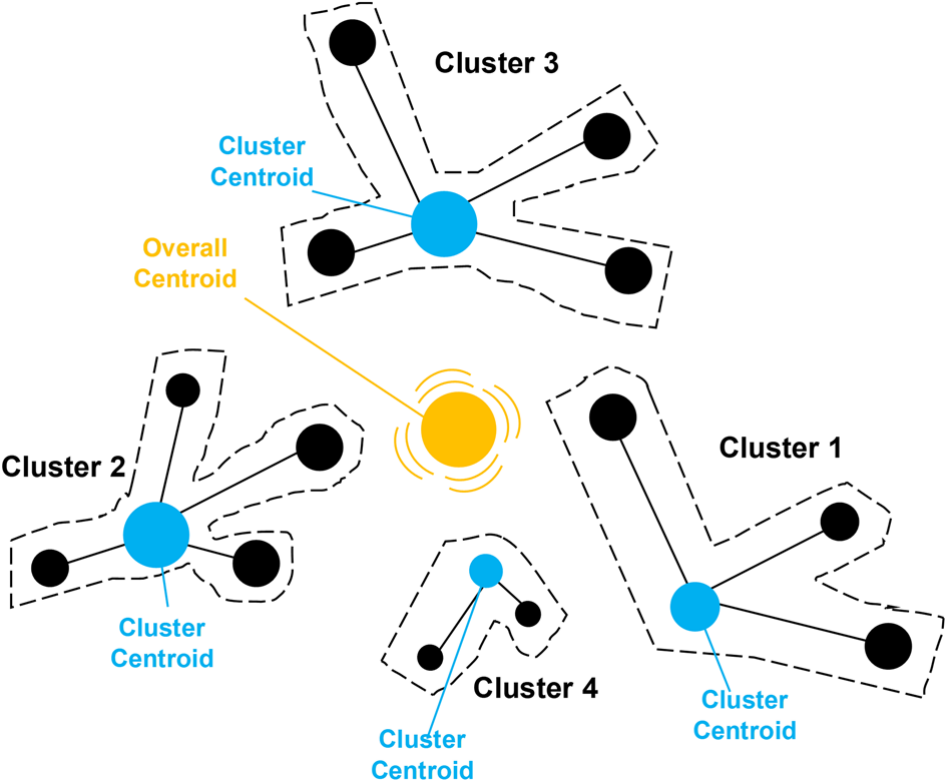
Illustration of Ward’s method

The objective function for the inner GA layer is based on Ward’s hierarchical method. This method minimises the intra-cluster distance (inside the cluster) while maximising the inter-cluster distance (between clusters). Ward’s method mainly relies on variance analysis, where the ultimate objective is to reduce the variance within the clusters. One of the major inconveniences of hierarchical methods, such as Ward’s method, is that it gravitates towards breaking the resulting clusters into smaller ones [27]. However, this can be alleviated by using a custom mutation operator that allows the merging of neighbouring clusters. Ward’s method is illustrated in Fig. 2 below.

To evaluate Ward’s outcome and determine the fitness value for each chromosome, we calculated two values, $\alpha$ and $\gamma$, which represent the inter-clustering and intra-clustering distances, respectively.

2 $$\begin{aligned} \mathbf {\alpha }_{\mathbf {c}}=\sum _{n=1}^{\mathrm {l}_c}{D\left( \bar{F_n},{\hat{F}}\right) ^2.S_n} \end{aligned}$$

where $\mathrm {l}_c$ is the number of clusters inside the chromosome at location c. $\bar{F_n}$ is the mean value of the features inside the cluster at location n. ${\hat{F}}$ is the centre of mass of the chromosome at location c. $D\left( \bar{F_n}, {\hat{F}}\right) ^2$ is the sum of squared differences (SSD) of the selected samples’ features, and $S_n$ is the size of the cluster at location n.

3 $$\begin{aligned} \gamma _c = \sum _{h=1}^{l_c}\frac{1}{2S_h}\sum _{F_n\in C_h}^{}\sum _{F_m\in C_h}^{} D(F_n, F_m)^2 \end{aligned}$$

where $\mathrm {l}_c$ is the number of clusters inside the chromosome at location c. $S_h$ is the size of the cluster at location h. $C_h$ is the samples inside the cluster at location h. $D\left( F_m,\ \bar{F_n}\right) ^2$ is the SSD of selected samples’ features. From the above equations, $\alpha _c$ reflects the distance and separation between the different clusters, while $\gamma _c$ measures the proximity of data inside the same cluster. So, the objective is to increase the value of $\alpha _c$ and decrease this of $\gamma _c$. The Calinski-Harabasz index was used to achieve this:

4 $$\begin{aligned} F_{CH} = \frac{\alpha _c}{\gamma _c} \times \frac{N-K}{K-1} \end{aligned}$$

where N is the number of clusters inside the chromosome at location c, and K is the number of samples. $F_{CH}$ is the fitness value for the chromosome, where a higher value means a better fitness, which translates to superior cluster separation with better feature selection and less noise.

As for the crossover operator, we used a modified version of a subtype of the edge recombination family, known as the maximal perseverance operator (MPX), which was initially suggested by Mühlenbein et al. [28]. This operator was used to explicitly transfer all the edges (loci) of the selected parents to their offspring while still maintaining its ability to generate new edges independently. Thus, this operator was used to preserve the original relation between the clustering groups while still allowing the forging of new local edges. This way, clustering configurations created by each chromosome would not be wiped out with a new generation.

To explain how the MPX operator works, we first select two parents according to the selection operator and then remove a random substring from the first parent. We then remove the remaining items (from the first parent) from the second parent. The items that remain from the second parent are then sequentially added to the first parent. Hence, we can guarantee that the same parents can generate many unique offspring, which are subjected to the selected substring’s location and size.

**Fig. 3:**
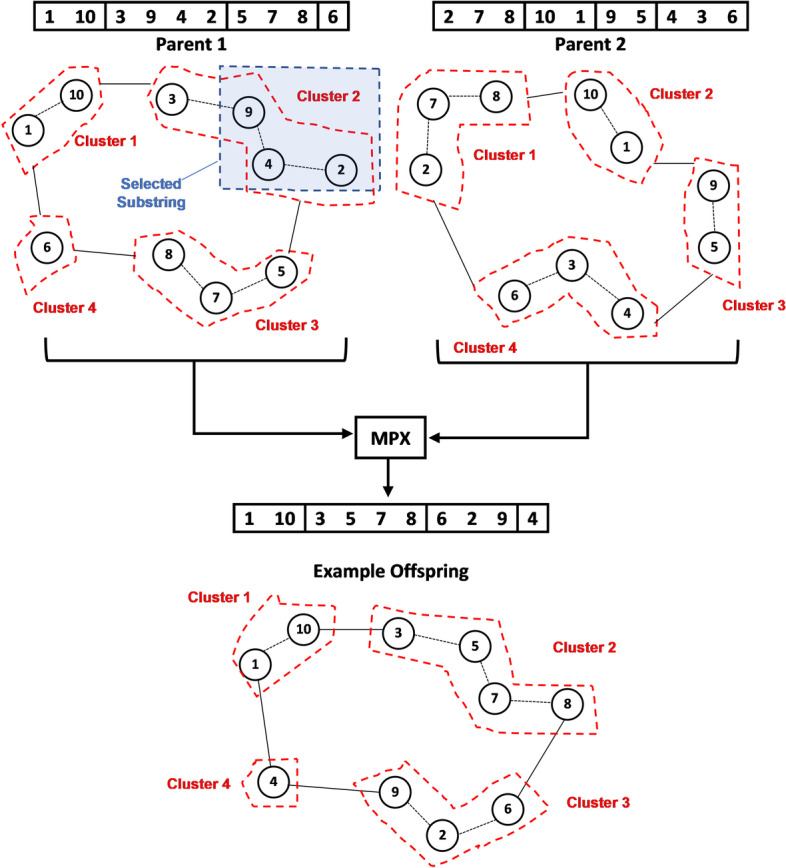
Illustration of the MPX operator

Since the intent is to maximise the distance between the clusters, the modified MPX operator was designed to target the selection of clusters with the shortest distance from the overall centroid. Subsequently, a random cut-off value relative to the cluster and a random size was calculated. Therefore, the MPX operator can totally or partially replace the selected cluster and substitute it with the cluster generated by the other parent. The crossover probability was set at 30%. Fig. 3 shows an illustration for the MPX operator. The second cluster is chosen in the example because it is assumed to have minimum inter-cluster distance relative to the first parent. Subsequently, the cut-off index and size were selected as 2 and 3, respectively. Therefore, the substring [9,4,2] was selected and removed from the first parent. The remaining items [1, 10, 3, 5, 7, 8, 6] were removed from the second parent. Then, the remaining items were consecutively inserted into the first parent. This process is done for the two selected parents and the offspring with the highest fitness value is selected, while the other is discarded.

Elitism was also implemented for the inner GA layer. This is important to assure that the fittest inner layer chromosome matches the corresponding outer layer chromosome and maintains the achieved clustering configuration throughout the convergence of the outer GA layer. A Roulette-wheel operator was used for selection. As for the mutation, a non-uniform operator similar to the one in the outer GA layer was implemented. However, the mutation operator in the inner GA layer works by either splitting or merging neighbouring clusters. The probability of the mutation operator increased with GA convergence and could reach a peak value of 10%. It ensured that the GA would not fall into a local-minimum clustering phenomenon during the convergence process.

### Supervised deep learning classification

In this stage, a deep neural network (DNN) classification model was implemented to use the features extracted from the previous metaheuristic stage. For this stage, two deep learning models are trained. The first is a binary classification model that combines the extracted features from the different cancer types to classify malignancy in samples, and the second model is a multi-classifier system that implements pan-classification for selected cancer diseases, using the common features.

Adam optimisation [29] was used to build the DNN models and accelerate training. Adam optimisation combines the root mean square propagation (RMSprop) and Stochastic Gradient Descent (SGD) with momentum descendant. It is accomplished using two estimations of moments (first and second) to readjust the learning rate. These two moments are the mean and the uncentered variance.

5 $$\begin{aligned} m_t=\beta _1m_{t-1}+(1-\beta _1)g_t \end{aligned}$$

6 $$\begin{aligned} v_t=\beta _2v_{t-1}+(1-\beta _2)g_t^2 \end{aligned}$$

where $m_t$and $v_t$ are moving averages and $g_t$ is the gradient of the current mini-batch. $\beta _1$and $\beta _2$ correspond to the exponential decay for the first and second moments estimate. Adam also implements epsilon, an extremely small number that prevents zero-division during the calculations.

We used a Multilayer Perceptron (MLP) with three hyperparameters to construct the neural network. $\beta _1$, $\beta _2$ and epsilon were retained at default values (0.9, 0.999 and 1e-08), as recommended by the original authors. The other hyperparameters (learning rate, hidden layer size, and the number of hidden layers) were optimised using different tests. Three learning rates (0.1, 0.01 and 0.001), four hidden layer sizes (512, 256, 128 and 64) and three hidden layer counts (1, 2, 4) were considered.

**Fig. 4:**
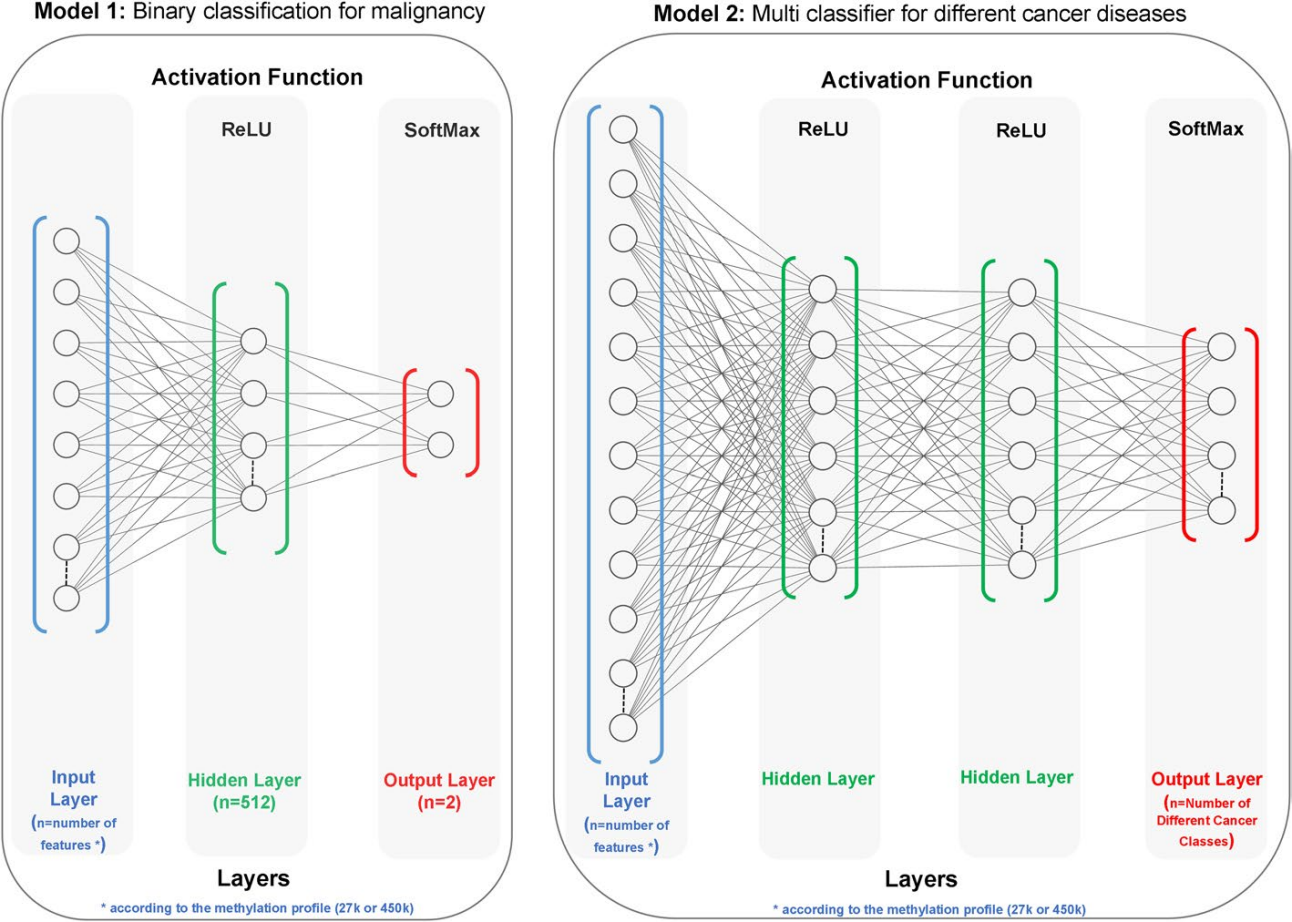
Schematic illustration of the two DNN architectures

We used a grid search technique [30] to select the optimum hyperparameter values. The best combination according to the evaluation set was (learning rate = 0.001, hidden layer size = 512 and the number of hidden layers = 1) for the first model, and (learning rate = 0.001, hidden layer sizes=(256,128) and the number of hidden layers = 2) for the second model. A rectified linear unit (ReLU) activation function was used for all models. Fig. 4 shows a schematic illustration for the DNN architectures.

The input layers have been set to accept the maximum number of CpG features according to the DNA profiling platform (27,578 for 27k profiles and 485,764 for the 450k profiles). Unselected features were assigned zero weights. The output classifiers for the binary classification model are malignant and benign. As for the pan-classification model, the output classifiers represent all the selected cancer diseases and a normal (benign) output. All the hidden layers are dense, which means that each neuron receives input from all neurons of its previous layer.

### Software application framework

This subsection describes the functionality of the application software used to implement the proposed system. An application, MetaMethyLib, was developed to implement the proposed system. The application was developed using the .NET framework and the C# programming language.

The software application starts by retrieving the DNA methylation data from TCGA according to the selected cancer disease and the tissue type (malignant or benign). It proceeds with data pre-processing and building the dataset for each cancer type. The application software was designed using a modular architecture. Each module performed a specific task, such as the data retrieval and processing, the nested GA implementation, and deep neural network implementation.

The dataset is expected to be very big and the literature review shows that most systems suffer from low performance with such enormous data sizes. Hence, the application software was developed using a parallel-processing architecture, using the .NET task parallel library. It allows the independent convergence of each outer layer chromosome for every generation using a logical CPU core to achieve maximum performance. Moreover, network convergence was implemented to increase the performance and scalability of the application software and its ability to handle more extensive datasets. Hence, the system can divide the GA convergence process over a group of connected clients where the primary host manages the entire process of executing and terminating the workload assigned to each client. For convenience, network clients can join or leave the workload queue at any time during GA convergence. It is important to note that the clients participating in the convergence process must have access to the dataset. Otherwise, the primary host will have to send the data remotely, which would result in excessive delays.

The host monitors the workload sent to each network client and maintains an ongoing connection. If a network client disconnects or stops responding, the host will resend the data to another client or choose to evolve it locally, depending on the workload queue. It is important to note that such implementation was only possible due to the proposed system architecture, which allows it to evolve with the help of other network clients. Each outer layer chromosome can be converged individually, and the results of each outer generation are forgathered by the host, before GA evolving operators like crossover or mutation are executed.

**Fig. 5:**
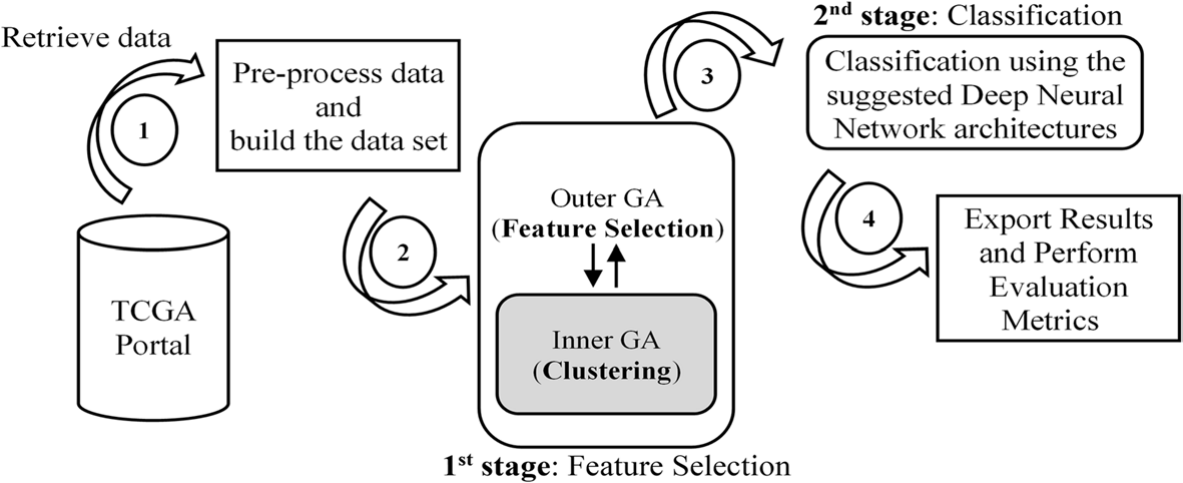
Workflow for the developed application software

As for the DNN classification stage, TensorFlow 2 using Keras.Net was used to build an addon module that implements the proposed architectures. The system was designed to run seamlessly on both the CPU and GPU. Fig. 5 shows the application software workflow.

## Experimental results and discussion

The developed application software processed the datasets to handle the missing methylation values using simple linear regression. However, samples with more than 10% and 20% (27k DNA profile and 450k DNA profile, respectively) missing features were rejected entirely from the final dataset. We adopted accuracy, precision, recall and Matthews Correlation Coefficient (MCC) for performance evaluation.

Recall measures the ability of the DNN to find all the positive samples, while the precision reflects the ability of the classifier not to label a sample as positive when it is negative.

MCC is a vigorous coefficient that sums up the classifier performance using −1 to +1 values, where +1 indicates a perfect classifier. We included the MCC metric because it has shown superiority with bioinformatics applications and generates a more insightful response than other metrics [31]. The formulae for these metrics are shown below:

7 $$\begin{aligned} Accuracy=\ \frac{TP+TN}{TP+TN+FP+FN} \end{aligned}$$

8 $$\begin{aligned} Recall=\ \frac{TP}{TP+FN} \end{aligned}$$

9 $$\begin{aligned} Precision=\ \frac{TP}{TP+FP} \end{aligned}$$

10 $$\begin{aligned} MCC=\ \frac{\left( TP.TN\right) -(FP.FN)}{\sqrt{\left( TP+FP\right) .\left( TP+FN\right) .\left( TN+FP\right) .(TN+FN)}} \end{aligned}$$

Where TP and TN values represent the correct predictions by the DNN, while FP and FN are the erroneous predictions for all samples.

### Feature selection

The DNA methylation datasets were divided into two random groups. The first group encompassed 70% of the samples and was used by the feature selection technique, and later on, to train the MLP DNN. The second group contained 30% of the samples and was used in the second stage to test the MLP DNN. The technique was tested five times before the results were averaged. The outer layer GA was set to run for 100 generations, while the inner layer GA was set to run for 200 generations for each outer layer chromosome. Since GA is a stochastic probabilistic technique, statistical methods were used to confirm the precision of the experiments.

All the experiments were executed on a high-performance computing (HPC) unit with an Intel Cascade Lake processor with 16 virtual central processing units (CPU) and 64GB of random-access memory (RAM). The HPC was also equipped with an NVIDIA Tesla P4 GPU. This HPC unit served as the primary host, while 16 other processing units, each with an Intel i7 9750H CPU and 16GB of RAM, served as network clients. After the system completed its convergence, The suggested feature selection technique successfully reduced data dimensionality for all cancer types.

**Fig. 6:**
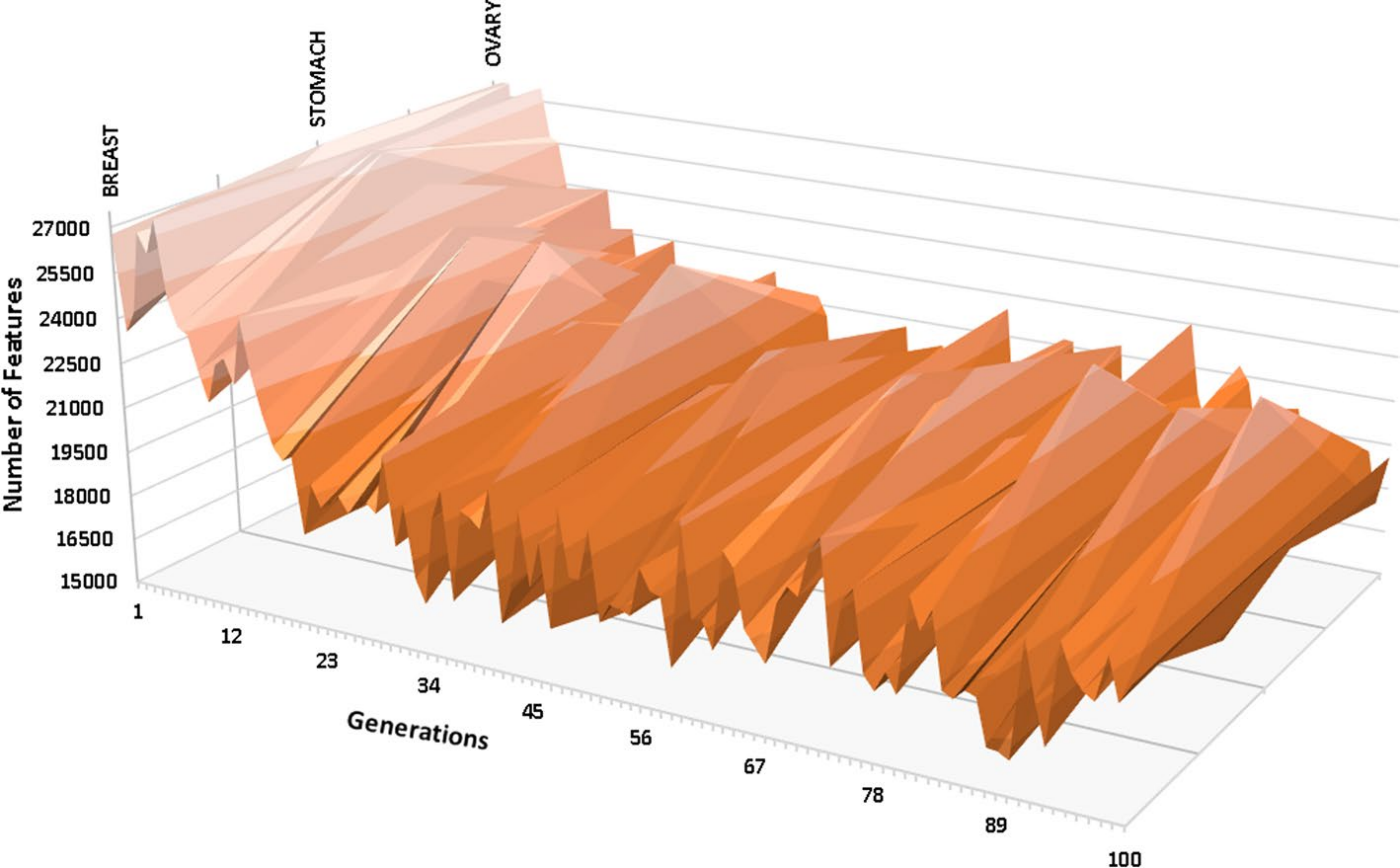
Surface plot showing data dimensionality changes for the 27k DNA methylation profile dataset

Table 3 shows the data dimensionality reduction for the 27k DNA methylation profile dataset, and Fig. 6 shows the change in the data dimensionality throughout the convergence process.

**Table 3:** Data dimensionality reduction for the 27k DNA methylation profile

Cancer name	Number of selected features
Breast	18,904 ($\sim$31.5% reduction)
Ovary	17,380 ($\sim$37% reduction)
Stomach	19,707 ($\sim$28.6% reduction)

The normalised 95% confidence interval for all the selected cancer types was $\pm$0.48%, $\pm$0.24% and $\pm$0.077% for Breast, Ovary and Stomach, respectively.

The number of common features (CpG loci) extracted from all the tested cancer diseases was 3,391.

**Fig. 7:**
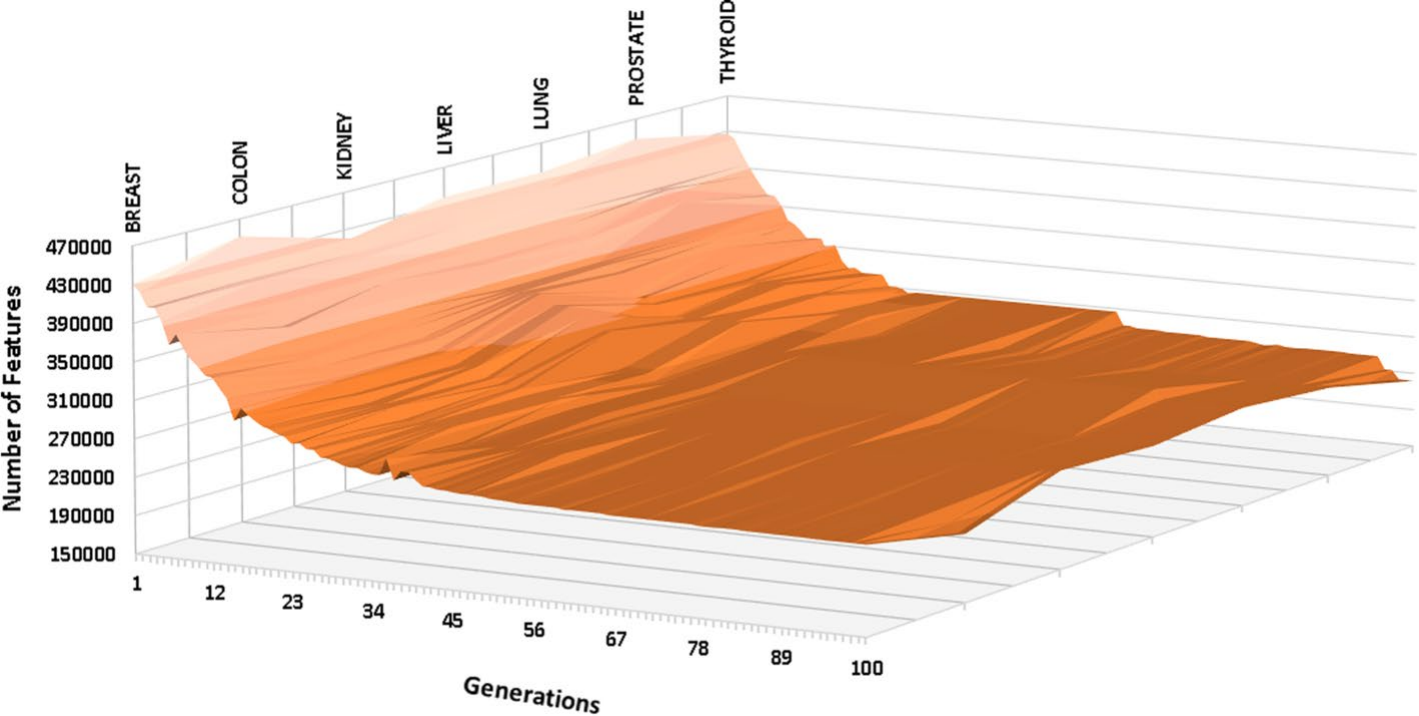
Surface plot showing data dimensionality changes for the 450k DNA methylation profile dataset

Table 4 shows the data dimensionality reduction for the 450k DNA methylation profile dataset, while Fig. 7 shows the change in the data dimensionality throughout the convergence process.

**Table 4:** Data dimensionality reduction for the 450k DNA methylation profile

Cancer name	Number of selected features
Breast	242,978 ($\sim$50.0% reduction)
Colon	220,111 ($\sim$65.0% reduction)
Kidney	252,373 ($\sim$48.0% reduction)
Liver	245,703 ($\sim$49.5% reduction)
Lung	254,645 ($\sim$47.6% reduction)
Prostate	245,042 ($\sim$50.0% reduction)
Thyroid	221,770 ($\sim$54.4% reduction)

For the 450k DNA methylation profile, the normalised 95% confidence interval for all the selected cancer types was $\pm$1.22%, $\pm$0.71%, $\pm$0.84%, $\pm$0.34%, $\pm$0.66%, $\pm$0.94% and $\pm$1.13% for Breast, Colon, Kidney, Liver, Lung, Prostate and Thyroid, respectively. The number of common features (CpG loci) extracted from all the tested cancer diseases was 4,273. The extracted common features will be applied on the input during the classification stage.

### DNN pan-classification

As previously stated, the DNA methylation datasets were randomly divided into two groups for training and testing the MLP DNN. The training group encompassed 70% of the dataset, and the testing group contained 30%.

Tables 5 and 6 show the number of samples used to train and test the system for the 27k DNA methylation profile dataset and the 450k DNA methylation profile dataset, respectively.

**Table 5:** Dataset (27k DNA Methylation profile) used to train and test the DNN models

Dataset	Total number of samples	Accepted samples	Training group (70%)	Testing group (30%)
Breast Cancer (BRCA)	342	341	237	104
Ovary Cancer (OV)	613	567	396	171
Stomach Cancer (STAD)	73	72	49	23

**Table 6:** Dataset (450k DNA Methylation profile) used to train and test the DNN models

Dataset	Total number of samples	Accepted samples	Training group (70%)	Testing group (30%)
Breast Cancer (BRCA)	596	584	408	176
Colon Cancer (COAD)	344	341	238	103
Kidney Cancer (KIRC)	439	433	303	130
Liver Cancer (LIHC)	258	226	158	68
Lung Cancer (LUSC)	291	286	200	86
Prostate Cancer (PRAD)	399	394	275	119
Thyroid Cancer (THCA)	454	454	317	137

**Fig. 8:**
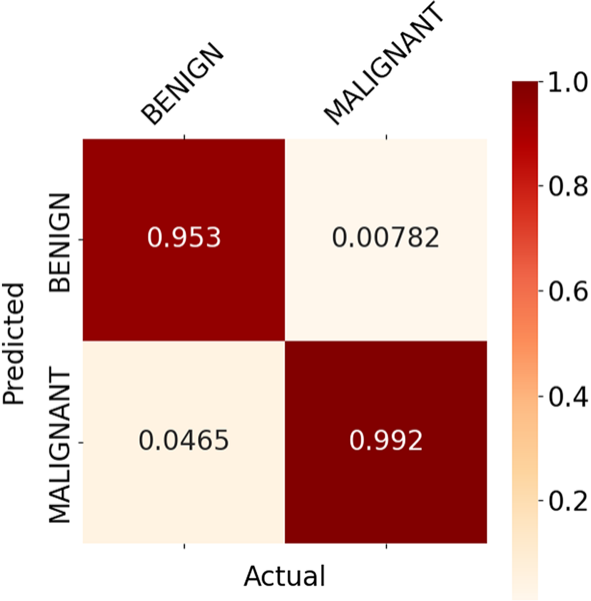
Normalised heat map confusion matrix of the binary classification DNN model, for the 27k DNA methylation profile dataset

**Fig. 9:**
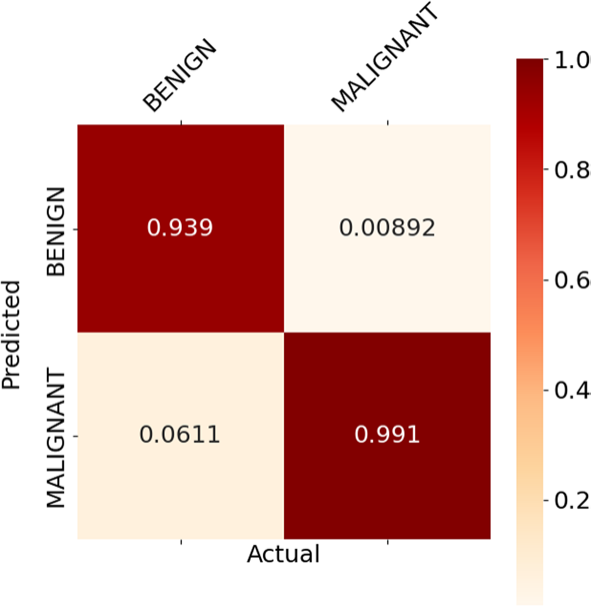
Normalised heat map confusion matrix of the binary classification DNN model, for the 450k DNA methylation profile dataset

For the binary classification model, the malignant groups for each cancer type were aggregated together, and the same was done for the benign groups. Figs. 8 and 9 below show the normalised heat map confusion matrix for the binary classification model for both DNA methylation profiles (27k and 450k).

**Fig. 10:**
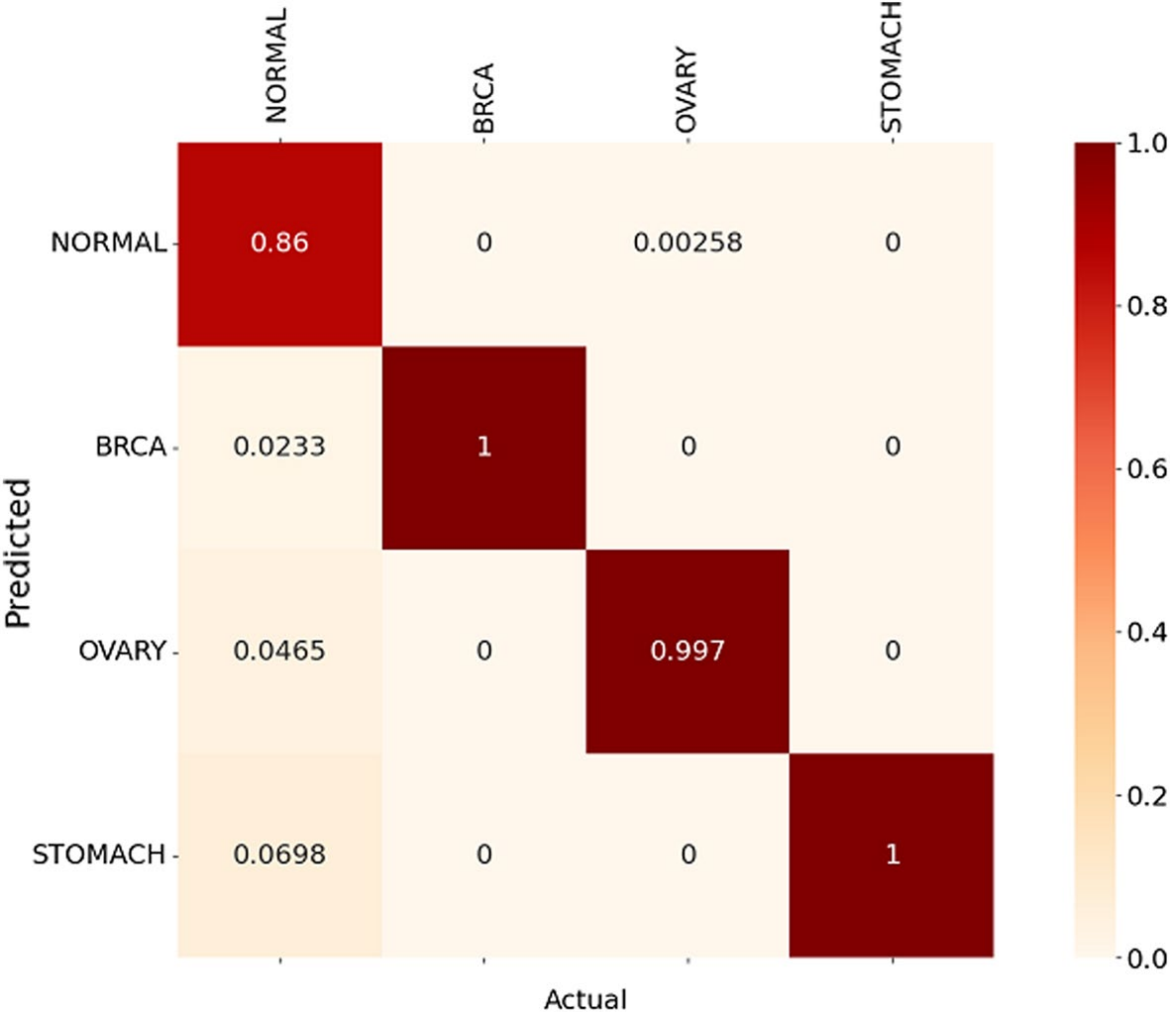
Normalised Heat map confusion matrix of the pan-classification DNN model, for the 27k DNA methylation profile dataset

**Fig. 11:**
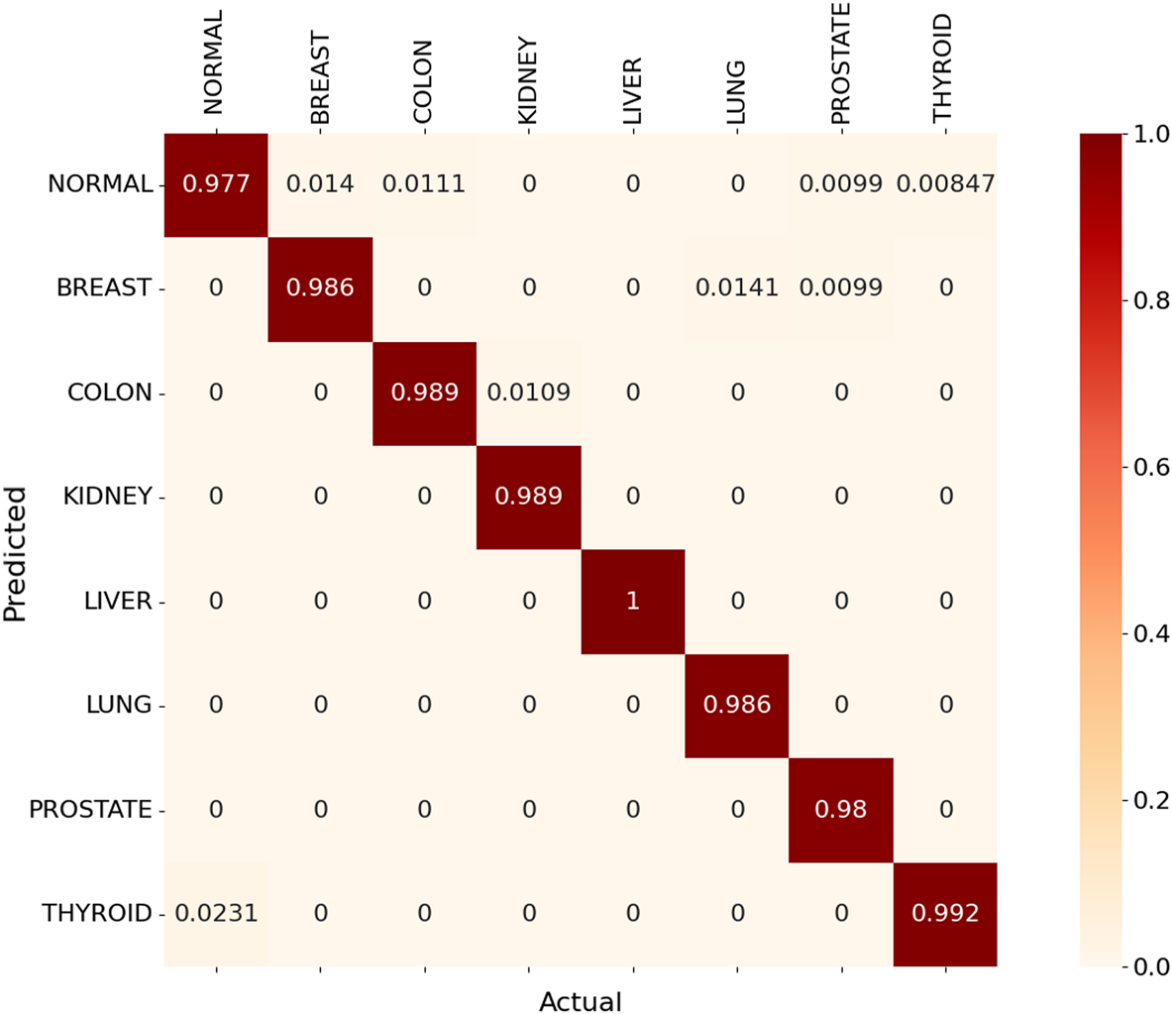
Normalised Heat map confusion matrix of the pan-classification DNN model, for the 450k DNA methylation profile dataset

Figs. 10 and 11 show the normalised heat map confusion matrix for the pan-classification DNN model for both DNA methylation profiles as well.

Tables 7 and 8 show the calculated performance metrics of the binary classification models for both DNA methylation profiles.

**Table 7:** Performance metrics of the binary classification model for the 27k DNA methylation profile

Performance metric	Tumour state	
	Benign (Normal)	Malignant (Cancer)
Accuracy	0.953	0.992
Precision	0.891	0.996
Recall	0.953	0.992
MCC	+0.916

**Table 8:** Performance metrics of the binary classification model for the 450k DNA methylation profile

Performance metric	Tumour state	
	Benign (Normal)	Malignant (Cancer)
Accuracy	0.939	0.991
Precision	0.953	0.988
Recall	0.938	0.991
MCC	+ 0.935	

The results are compared with the recent work by Zheng et al. [17] and Modhukur et al. [18] for the compatible cancer types from primary sources.

The MCC values of the pan-classification DNN models were +0.981 and +0.984 for the 27k DNA methylation profile and the 450k DNA methylation profiles, respectively.

For the first study (pilot) that uses the 27k DNA methylation profile dataset, the proposed method outperformed the other two methods and achieved higher precision and accuracy for the breast cancer (BRCA).

For the stomach cancer(STAD), the proposed technique outperformed the DNN technique proposed by Zheng et al. in terms of precision and recall, and achieved better recall compared to the RF method proposed by Modhukur et al. As for ovary cancer(OV), the system was able to achieve results that are very similar to the RF method. As for the results of the second study that uses the 450k DNA methylation profile dataset, the proposed method outperformed the other two methods and achieved higher precision and accuracy for colon(COAD), liver(LIHC) and lung(LUSC) cancers. The proposed technique was able to outperform the RF method in terms of precision and recall for breast (BRCA), kidney (KIRC) and prostate (PRAD) cancers. For the thyroid cancer (THCA), the proposed system achieved a higher recall value compared with the DNN method and close results compared to the RF method. Tables 9 and 10 highlight the results of the pan-classification DNN models for both DNA methylation profiles, based on the precision and recall metrics. Tables 11 and 12 present the accuracy and F1-score metrics of the proposed system for both DNA methylation methods, respectively.

**Table 9:** Performance comparison (precision and recall) with recent methods based on the first study with the 27k DNA methylation profile dataset

Cancer type	Method	Precision	Recall
Breast Cancer (BRCA)	DNN (Zheng et al.)	0.9810	1.00
	RF (Modhukur et al.)	0.976	0.932
	Proposed method	0.995	1.00
Stomach Cancer (STAD)	DNN (Zheng et al.)	0.8721	0.9375
	RF (Modhukur et al.)	0.944	0.964
	Proposed method	0.9142	1.00
Ovary Cancer (OV)	DNN (Zheng et al.)	–	–
	RF (Modhukur et al.)	1.0	1.0
	Proposed method	0.994	0.997

**Table 10:** Performance comparison (precision and recall) with recent methods, based on the second study with the 450k DNA methylation profile dataset

Cancer type	Method	Precision	Recall
Breast Cancer (BRCA)	DNN (Zheng et al.)	0.981	1.0
	RF (Modhukur et al.)	0.976	0.932
	Proposed method	0.986	0.986
Colon Cancer (COAD)	DNN (Zheng et al.)	–	–
	RF (Modhukur et al.)	0.993	0.980
	Proposed method	0.988	0.988
Kidney (KIRC)	DNN (Zheng et al.)	1.0	1.0
	RF (Modhukur et al.)	1.0	0.974
	Proposed method	1.0	0.99
Liver (LIHC)	DNN (Zheng et al.)	0.66	1.0
	RF (Modhukur et al.)	1.0	0.99
	Proposed method	1.0	1.0
Lung (LUSC)	DNN (Zheng et al.)	1.0	0.66
	RF (Modhukur et al.)	0.91	0.953
	Proposed method	1.0	0.986
Prostate (PRAD)	DNN (Zheng et al.)	1.0	1.0
	RF (Modhukur et al.)	1.0	0.980
	Proposed method	1.0	0.980
Thyroid (THCA)	DNN (Zheng et al.)	1.0	0.987
	RF (Modhukur et al.)	1.0	1.0
	Proposed method	0.975	0.99

**Table 11:** Performance comparison (f1-score and accuracy) with the RF method, based on the first study with 27k DNA methylation profile dataset

Cancer type	Method	F1-Score	Accuracy
Breast Cancer (BRCA)	RF (Modhukur et al.)	0.954	0.926
	Proposed method	0.997	1.0
Stomach Cancer (STAD)	RF (Modhukur et al.)	0.954	0.939
	Proposed method	0.955	1.0
Ovary Cancer (OV)	RF (Modhukur et al.)	1.0	1.0
	Proposed method	0.996	0.997

**Table 12:** Performance comparison (f1-score and accuracy) with the RF method, based on the second study with 450k DNA methylation profile dataset

Cancer type	Method	F1-score	Accuracy
Breast Cancer (BRCA)	RF (Modhukur et al.)	0.954	0.926
	Proposed method	0.986	0.986
Colon Cancer (COAD)	RF (Modhukur et al.)	0.987	0.980
	Proposed method	0.988	0.989
Kidney Cancer (KIRC)	RF (Modhukur et al.)	0.987	0.993
	Proposed method	0.99	0.989
Liver Cancer (LIHC)	RF (Modhukur et al.)	0.99	0.99
	Proposed method	1.0	1.0
Lung Cancer (LUSC)	RF (Modhukur et al.)	0.931	0.951
	Proposed method	0.99	0.986
Prostate Cancer (PRAD)	RF (Modhukur et al.)	0.98	0.98
	Proposed method	0.99	0.98
Thyroid Cancer (THCA)	RF (Modhukur et al.)	1.00	0.99
	Proposed method	0.98	0.99

It is important to note that the importance of these performance metrics must be interpreted according to the target application. In the case of cancer (or any other disease), false negatives must be averted since they can lead to lethal outcomes. In other words, recall is much more important than precision in this case.

Another very popular metric that represents the accuracy of the proposed system, at different threshold values, is the receiver operating characteristic (ROC) curves and their respective areas [32].

**Fig. 12:**
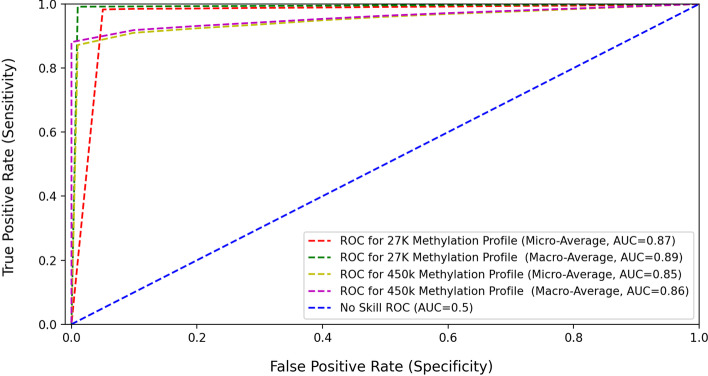
ROC plot for the pan-classification model

The ROC curves for the pan classification model are shown in Fig. 12. These curves can help visualize how well the classification model is performing. ROC works by plotting the true positive rate (TPR), also known as sensitivity, against the false positive rate (FPR), known as specificity.

Since we are calculating the AUC for a multi-class model, two values: macro-average and micro-average, were calculated.

The macro-average computes the metric for each class independently, then averages the resulting values, hence treating all classes equally.

Whereas a micro-average combines the contribution of all classes to calculate the mean metric, taking into consideration the imbalance in the number of samples. The area under the curve (AUC) for ROC reflects the ability of the classifier to distinguish between the different classes.

When the value of AUC is equal to 1, this means that the classification model can perfectly distinguish between all various classes correctly.

An AUC value between 0.5 and 1 indicates a higher probability that the classification model will be able to distinguish the positive class values from the negative ones. A value of 0.5 for AUC (e.g., 45 degrees diagonal line) means that the classification model has no discriminatory ability (no skill).

The calculated AUC values of the pan classification model for the first study (27k DNA methylation profile)were 0.87 and 0.89 for the micro-average and macro-average, respectively.

As for the second study (450k DNA methylation profile), the AUC values of the pan classification model were 0.85 and 0.86 for the micro-average and macro-average, respectively. An AUC of 0.6 to 0.7 is considered poor, 0.7 to 0.8 is considered good and 0.8 to 0.9 is considered excellent [33].

Finally, it is noteworthy to highlight that current research shows evidence that some DNA methylation markers derived from blood can mimic DNA methylation signatures found in internal tissues from primary cancer sources [34]. Therefore, this research can potentially help identify early-stage cancer by testing blood-derived samples using the proposed classification model.

## Conclusion

This research presented an effective new approach to classify different cancer types based on DNA methylation data retrieved from TCGA. The system first used a metaheuristic technique to perform feature extraction to decrease data dimensionality. It then implemented deep neural network techniques to classify different cancer diseases. The importance of using a metaheuristic technique is indicated by its ability to discover relevant hidden patterns and substantial information in the data, especially since, as stated in the literature, the biological process of DNA methylation is still not completely understood.

An initial pilot study using three cancer types (breast, stomach and ovary) that were sampled using the Illumina Infinium 27k DNA methylation platform, was conducted. Then to test the scalability and performance of the proposed method, another study that encompasses other major cancer types (colon, kidney, liver, lung, prostate and thyroid) that were sampled using the superior Illumina Infinium 450k DNA methylation platform, was conducted.

The performance of the proposed system was compared to recently published works. It provided better results for most cancer types, confirming the effectiveness of of the proposed system for classifying different cancer types based on DNA methylation data (Additional files 1, 2, 3, 4, 5, 6, 7, 8, 9 and 10).

## Supplementary information

**Additional file 1:** Metadata file containing ID(s) and information about the 27k methylation platform breast cancer samples, created JAN 2019.

**Additional file 2:** Metadata file containing ID(s) and information about the 27k methylation platform ovary cancer samples, created JAN 2019.

**Additional file 3:** Metadata file containing ID(s) and information about the 27k methylation platform stomach cancer samples, created JAN 2019.

**Additional file 4:** Metadata file containing ID(s) and information about the 450k methylation platform breast cancer samples, created APRIL 2022.

**Additional file 5:** Metadata file containing ID(s) and information about the 450k methylation platform colon cancer samples, created APRIL 2022.

**Additional file 6:** Metadata file containing ID(s) and information about the 450k methylation platform kidney cancer samples, created APRIL 2022.

**Additional file 7:** Metadata file containing ID(s) and information about the 450k methylation platform liver cancer samples, created APRIL 2022.

**Additional file 8:** Metadata file containing ID(s) and information about the 450k methylation platform lung cancer samples, created APRIL 2022.

**Additional file 9:** Metadata file containing ID(s) and information about the 450k methylation platform prostate cancer samples, created APRIL 2022.

**Additional file 10:** Metadata file containing ID(s) and information about the 450k methylation platform thyroid cancer samples, created APRIL 2022.

## Data Availability

The samples used for this research can be found in the Genomic Data Commons (GDC) Data Portal https://portal.gdc.cancer.gov/. The ID(s) of all samples that were used in this research are attached with the article (https://github.com/smnoureldini/MetaMethyLib).

## Abbreviations

TCGA: The cancer genome atlas
GA: Genetic algorithm
ILGA: Inner layer genetic algorithm
OLGA: Outer layer genetic algorithm
MPX: Maximal perseverance operator
DNN: Deep neural network
MLP: Multilayer perceptron
ROC: Receiver operating characteristic
AUC: Area under the receiver operating characteristic curve
